# The connexin43 mimetic peptide Gap19 inhibits hemichannels without altering gap junctional communication in astrocytes

**DOI:** 10.3389/fncel.2014.00306

**Published:** 2014-10-21

**Authors:** Verónica Abudara, John Bechberger, Moises Freitas-Andrade, Marijke De Bock, Nan Wang, Geert Bultynck, Christian C. Naus, Luc Leybaert, Christian Giaume

**Affiliations:** ^1^Center for Interdisciplinary Research in Biology, Centre National de la Recherche Scientifique, Collège de FranceParis, France; ^2^Department of Cellular and Physiological Sciences, Faculty of Medicine, Life Sciences Institute, University of British ColumbiaVancouver, BC, Canada; ^3^Department of Basic Medical Sciences - Physiology Group, Faculty of Medicine and Health Sciences, Ghent UniversityGhent, Belgium; ^4^Laboratory of Molecular and Cellular Signaling, Department of Cellular and Molecular MedicineKU Leuven, Leuven, Belgium

**Keywords:** connexins, glial cells, gap junctions, astroglia, mimetic peptide

## Abstract

In the brain, astrocytes represent the cellular population that expresses the highest amount of connexins (Cxs). This family of membrane proteins is the molecular constituent of gap junction channels and hemichannels that provide pathways for direct cytoplasm-to-cytoplasm and inside-out exchange, respectively. Both types of Cx channels are permeable to ions and small signaling molecules allowing astrocytes to establish dynamic interactions with neurons. So far, most pharmacological approaches currently available do not distinguish between these two channel functions, stressing the need to develop new specific molecular tools. In astrocytes two major Cxs are expressed, Cx43 and Cx30, and there is now evidence indicating that at least Cx43 operates as a gap junction channel as well as a hemichannel in these cells. Based on studies in primary cultures as well as in acute hippocampal slices, we report here that Gap19, a nonapeptide derived from the cytoplasmic loop of Cx43, inhibits astroglial Cx43 hemichannels in a dose-dependent manner, without affecting gap junction channels. This peptide, which not only selectively inhibits hemichannels but is also specific for Cx43, can be delivered *in vivo* in mice as TAT-Gap19, and displays penetration into the brain parenchyma. As a result, Gap19 combined with other tools opens up new avenues to decipher the role of Cx43 hemichannels in interactions between astrocytes and neurons in physiological as well as pathological situations.

## Introduction

Compared to neurons, astrocytes make up the brain cell population that expresses the highest amount of the gap junction proteins, named connexins (Cxs) (Ransom and Giaume, 2013). Connexin-mediated channel functions are essential for the dynamic and metabolic interactions that astrocytes establish with each other and at their interfaces with neurons and the vasculature (Giaume et al., 2010). Indeed, transgenic animals in which the two major astroglial Cxs, i.e., Cx43 and Cx30, have been deleted, exhibit impaired potassium clearance, synaptic transmission and plasticity (Wallraff et al., 2006; Pannasch et al., 2011), a dysmyelinating phenotype (Lutz et al., 2009) and a loss in blood-brain barrier integrity (Ezan et al., 2012). However, the exclusive use of such animals does not distinguish between the contributions of the two types of astroglial Cxs as well as between the channel and hemichannel functions that they support (Giaume and Theis, 2010). So far, the use of single Cx knock-out mice has provided key data demonstrating a role of Cx43 in neuronal migration (Elias et al., 2007; Cina et al., 2009), a synaptic activity-dependent modulation of Cx30 gap junctions in astrocytes in the olfactory bulb (Roux et al., 2011) and recently, it was reported that the lack of Cx30 impacts synaptic transmission through the modulation of astroglial glutamate transport (Pannasch et al., 2014). However, there is still a need to develop new pharmacological tools to design *in vitro* and *in vivo* experiments studying the role of Cxs in astrocytes.

**Figure 1:**
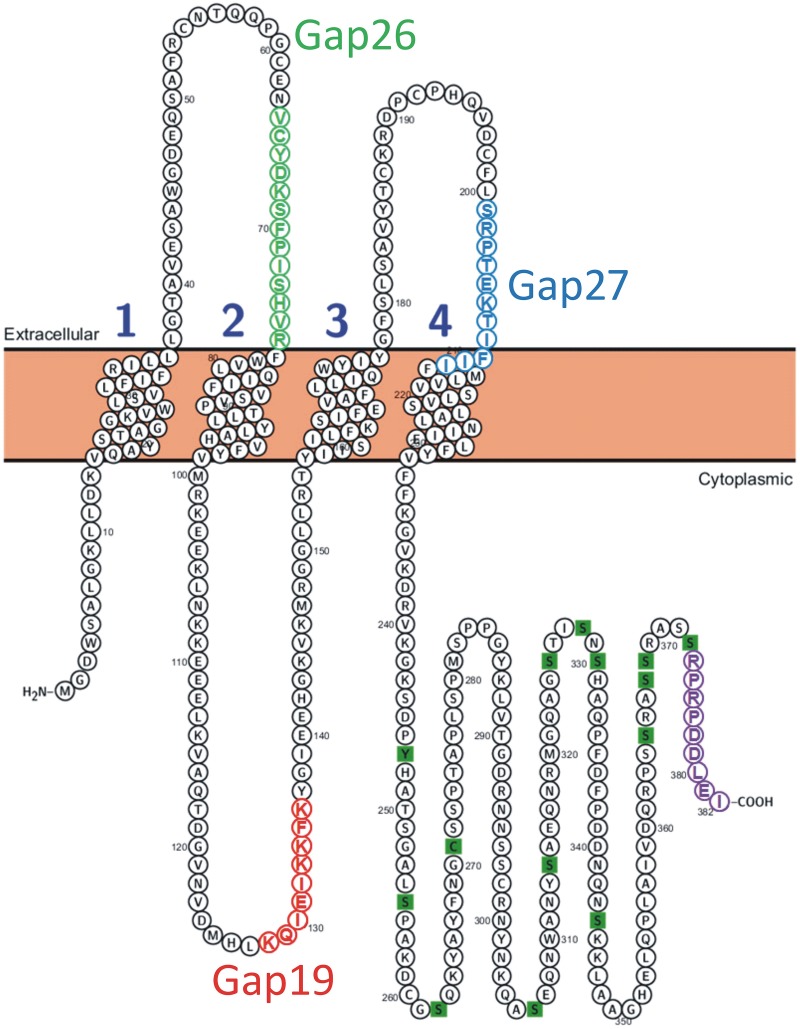
**Position of the Gap19 sequence in the intracellular cytoplasmic loop domain of human Cx43**. One identified interaction site is located in the last 9 AAs of the CT-tail marked in purple (Wang et al., 2013a). The sequences of Gap19 (red) on the intracellular loop, Gap26 (green) and Gap27 (blue) on the extracellular loops are indicated on the drawing. The CT residues marked as green squares are sites of posttranslational modifications and have been added for illustration purposes [Illustration generated with the Protter tool (Omasits et al., 2014)].

Gap junction channels form junctional plaques that are composed of two docked hemichannels oligomerized from six Cx protein subunits. Usually, most of the unapposed/non-junctional hemichannels are closed but a fraction of Cx43 HCs can be open under resting conditions and have physiological roles (Stehberg et al., 2012; Chever et al., 2014) while they become more active in pathological situations (Giaume et al., 2013). Their activation results in gliotransmitter (ATP, glutamate) release, the entry of calcium ions (Ca^2+^) and glucose, ionic imbalance, cellular volume overload and, in certain cases, cell death (Decrock et al., 2009; De Bock et al., 2013; Giaume et al., 2013). Currently, there are no tools available that allow selective targeting of hemichannels since all known pharmacological blockers, including glycyrrhetinic acid-derivatives such as carbenoxolone or related molecules with improved blood-brain barrier permeability (Takeuchi et al., 2011), poorly discriminate between gap junctions and hemichannels. Additionally, they mostly affect Cx channels composed of various distinct Cx types (Harris, 2001; Evans et al., 2006; Spray et al., 2006; Saez and Leybaert, 2014). Beside these derivatives of glycyrrhetinic acid, other compounds such as gadolinium (Gd^3+^) and lanthanum (La^3+^) are supposed to affect only hemichannels but, especially in the nervous system where neurons are present, they have side effects that limit the interpretation of their use. Connexins are tetraspan membrane proteins that have two extracellular (EL) loops and one intracellular cytoplasmic loop (CL). Synthetic peptides like Gap26 and Gap27 that mimic a short stretch of amino acids (AAs) on the extracellular loops have been developed more than two decades ago to inhibit gap junctional communication (Warner et al., 1995) (for Gap26 and Gap27 sequences see Figure 1). These peptides are thought to interact with the extracellular loops and block hemichannel activity within minutes (Wang et al., 2012; Giaume et al., 2013). They also prevent the docking of two facing hemichannels and thus affect gap junctional communication when applied for periods of several hours (Evans and Boitano, 2001; Decrock et al., 2009). Similarly, antibodies directed against the EL domains of the Cx protein rapidly inhibit hemichannels but they also display delayed inhibition of gap junction channels by preventing the processes of hemichannel docking and *de novo* gap junction channel formation (Orellana et al., 2011; Riquelme et al., 2013). In some cases, distinctive effects on hemichannels and gap junctions depend on the concentration at which they are applied: peptide5, which contains a sequence that comprises part of the Gap27 domain (SRPTEKT), inhibits hemichannels at low (5 μM) concentration while combined gap junction/hemichannel block is only observed at high (500 μM) concentration (O'carroll et al., 2008).

Here, we describe the effect on astroglial hemichannels of a peptide, named Gap19, that is identical to a short sequence present on the intracellular CL domain of Cx43 (Figure 1). Peptides mimicking CL sequences have been frequently used as control peptides for gap junction work, since it was shown that these do not inhibit gap junctional coupling (Evans and Leybaert, 2007). Although this specificity has already been reported for the cardiac system (Wang et al., 2013b) we think that it is important to validate this property for the nervous system. Indeed, this is particular relevant for the astrocytes because Cx43 hemichannels contribute to “gliotransmission” and can thus be involved in neuroglial interactions that participate to the control of neuronal activity and survival (see Giaume et al., 2013). In line with this, we found that Gap19 did not inhibit gap junction coupling in astrocytes as measured with dye transfer assays. At the same time, however, Gap19 strongly inhibited Cx43 hemichannels as exemplified by ATP release and dye uptake assays. Finally, we provide evidence that the TAT version of Gap19 is able to cross the intact blood-brain barrier indicating that this peptide can be used to block astroglial Cx43 hemichannel activity when applied through a vascular route.

## Materials and methods

### Animals

Cultures and slices were obtained from OF1, C57Bl/6 and GFAP-eGFP mice. All experiments were performed according to the European Community Council Directives of November 24, 1986 (86/609/EEC) and all efforts were made to minimize the number of animals used and their suffering. For the *in vivo* experiments with TAT-Gap19 all breeding and animal procedures were approved by The University of British Columbia Animal Care Committee or Ghent University Animal Experiment Ethical Committee and performed in accordance with the guidelines established by the Canadian Council on Animal Care or European Ethics Committee guidelines.

### Astrocyte cultures

Primary astrocyte cultures were prepared from the cortex of newborn (1–2 days) OF1 mice as described previously (Meme et al., 2006). Briefly, cells were seeded into 100-mm diameter plastic dishes (Nunc, Roskilde, Denmark) at the density of 3 × 10^6^ cells/dish in DMEM (Sigma-Aldrich, St-Louis MO, USA), supplemented with penicillin (5 U/ml), streptomycin (5 μg/ml) (Invitrogen, Carlsbad, CA, USA) and 10% FCS (Hyclone, Logan, UT, USA). The medium was changed twice a week. When cells reached confluence, around 10 days *in vitro* (DIV), they were harvested with trypsin-EDTA (Invitrogen). Then, cells were re-plated (2 × 10^5^ cells per well), as secondary cultures, on glass coverslips (Gassalem, Limeil-Brévannes, France) placed inside 16-mm diameter 4 well plastic plates for hemichannel assay and on 35 mm diameter petri dishes for scrape loading dye transfer technique. Finally, they were grown to confluence (about 1 week) and the medium was changed twice a week until the experiments were carried out.

### Acute hippocampal slices

GFAP-eGFP mice (Nolte et al., 2001) were decapitated and their brains were rapidly removed. Hippocampi were dissected and placed in ice-cold artificial cerebrospinal fluid (ACSF) equilibrated with 95% O_2_–5% CO_2_. Transverse hippocampal slices (300–400 μm thick) were cut on a vibroslicer (Leica VT 1000S, Wetzlar, Germany) and transferred to a holding chamber where they rested on a nylon mesh, submerged in oxygenated ACSF at room temperature for a stabilization period of 45 min before recodings. The ACSF solution contained in mM: NaCl 134; KCl 2.8; NaHCO_3_ 29; NaH_2_PO_4_ 1.1; glucose 12; MgSO_4_ 1.5; CaCl_2_ 2.5.

### Dye uptake experiments

Hemichannel activity in cultured astrocytes was induced either by treatment with two pro-inflammatory cytokines, TNF-α and IL-1β (Retamal et al., 2007) or by exposing the cells to a Ca^2+^-free solution (Stout et al., 2002). The hemichannel-permeable fluorescent tracer ethidium bromide (Etd^+^, 314 Da) was applied for 10 min at 4 μM final concentration and at 37°C. Then, cells were washed with Hank's balanced salt solution (HBSS) in mM: NaCl: 137; KCl: 5.4; Na_2_HPO_4_: 0.34; KH_2_PO_4_: 0.44, at pH 7.4 and supplemented with 1.2 mM CaCl_2_ (HBSS-Ca^2+^). Finally, astrocytes were mounted in Fluoromount and examined by epifluorescence (518 nm excitation and 605 nm emission) using an inverted microscope (Diaphot-Nikon, Nikon France S.A, Champigny sur Marne) equipped with a CCD camera (Nikon) associated with image analyzer software (Lucia-Nikon). Captured images of Etd^+^ uptake were analyzed with the Image J program (NIH software).

In slices derived from GFAP-eGFP mice, astrocytes were identified by GFP fluorescence and hemichannel activity was investigated. In this mouse, not all astrocytes are GFP-positive (Nolte et al., 2001; Houades et al., 2006) and Etd^+^ uptake was also observed in cells that were not positive for GFP. These cells could be GFP-negative astrocytes, neurons or microglial cells, in which hemichannel activity has been reported (see for instance Orellana et al., 2011). In order to be sure that we were quantifying Etd^+^ uptake in astrocytes, in this study hemichannel activity was only considered in GFP-positive cells. As previously described (Giaume et al., 2012), living slices were incubated with Etd^+^ for 10 min at room temperature and at 4 μM final concentration. Slices were also treated for 3 h with lipopolysaccharide (LPS, 1–100 ng/ml), a procedure know to activate microglial cells and induce Cx43 hemichannel activity in astrocytes (Retamal et al., 2007). Then, following the 10 min incubation with Etd^+^, slices were rinsed 15 min in ACSF to stop dye uptake and reduce background labeling before submerging them for 2 h in fixing solution (4% paraformaldehyde in 0.12 M buffer phosphate). Fixed slices were then rinsed in PBS and mounted in Fluoromount-G medium until photomicrographs were taken. Labeled cells were visualized with a 40x objective in a microscope equipped with epifluorescence illumination and appropriate filters for Etd^+^ (excitation wavelength, 528 nm; emission wavelength, 598 nm) and GFAP-eGFP (excitation wavelength, 488 nm; emission wavelength, 507 nm). Alternatively, immunolabeled astrocytes were examined at 63x and 20x with a confocal laser-scanning microscope (Leica TBCS SP2). Stacks of consecutive confocal images taken at 1 μm intervals were acquired sequentially with two lasers (argon 488 nm for GFAP-eGFP and 561 nm for Etd^+^). Fluorescence was quantified in arbitrary units (AU), Image J program (NIH software). Dye uptake intensity was evaluated as the difference (F—F_0_) between the fluorescence (F) from astrocytes (20—30 cells per slice) and the background fluorescence (F_0_) measured in the same field where no labeled cells were detected. At least three fields were selected in every slice for background evaluation.

### Determination of gap junctional communication

Experiments were performed by using the scrape loading dye transfer technique, as previously described (Meme et al., 2006). Cells were incubated at room temperature for 10 min in HEPES buffered salt solution containing (in mM): NaCl, 140; KCl, 5.5; CaCl_2_, 1.8; MgCl_2_, 1; glucose, 10; HEPES, 10 at pH 7.35. Cells were then washed in calcium-free HEPES solution for 1 min and the scrape loading and dye transfer assay (see Giaume et al., 2012) was carried out in the same calcium-free solution containing Lucifer yellow CH (427 Da, 1 mg/ml). After 1 min, cells were washed with the HEPES solution and Lucifer yellow loaded in the cells was allowed to diffuse through gap junction channels for 8 min. Photomicrographs were taken and data were quantified using NIS Nikon software. In all experiments, the fluorescence area of the first row of cells initially loaded, as measured in the presence of the gap junction channel inhibitor carbenoxolone (100 μM, 10 min), was subtracted from the total fluorescence area.

### TAT-Gap19 *in vivo* administration

Two different approaches were used for *in vivo* delivery of TAT-Gap19. For acute experiments, 4 months old C57Bl6 male mice were subjected to intra-carotid injection of 45 mg/kg TAT-Gap19 (GenScript, Pistcataway, NJ, US) dissolved in saline. After 1 h the mice were deeply anesthetized with sodium pentobarbital (120 mg/kg intraperitoneally) and were transcardially perfused with cold ice phosphate-buffered saline (PBS) followed by perfusion with 10% formalin (Sigma-Aldrich, Oakville, Canada). Brains were removed and stored in 10% formalin and the next day were cryoprotected in 30% sucrose in PBS solution. Cortical brain sections, 10 μm thick, were collected and mounted sequentially on glass microscope slides. Immunohistochemistry was performed on the sections as previously described (Ozog et al., 2002; Nakase et al., 2004; Kozoriz et al., 2010). Ten μm thick glass mounted sections were rehydrated in PBS and then blocked in PBS pH 7.4 containing 0.3% Triton-X-100 (TX100, Fisher Scientific) and 2% bovine serum albumin (BSA; Invitrogen, Canada) for 1 h, and incubated overnight in PBS containing 1.0% BSA, 0.3% TX100 and anti-TAT primary antibody (1/100 dilution; Cat # CB0888, Cell Applications, Inc., USA). The following day, the slices were washed with PBS (3 × 10 min) and subsequently incubated for 1 h with secondary antibody (anti-mouse IgG tagged with Alexa 488; Molecular Probes, USA) in PBS containing 1% BSA, 0.3% TX100. Slices were then rinsed 3 times for 10 min with PBS and mounted with ProLong Gold antifade reagent with DAPI. Images were obtained using the same exposure times on a Ziess Axiophot Epifluorescent microscope, Zeiss Canada, Toronto).

For experiments with i.v. administration, 55 mg/kg TAT-Gap19 was injected *via* the tail vein to obtain an estimated 250 μM concentration in the blood (assuming a blood volume of 8% of the body weight). Twenty-four hours after injection of the peptide, mice were deeply anesthetized by i.p. injection of ketamine (240 mg/kg) and xylazine (12 mg/kg) and were transcardially perfused with PBS. Brains were removed, snap-frozen in liquid nitrogen-cooled isopentane and mounted in cryoprotectant (Klinipath) before storage at −80°C. For staining, brains were sliced with a cryostat into 25 μm thick coronal sections, mounted on superfrost plus microscope slides (Thermo Scientific) and fixed in 4% paraformaldehyde (25 min, RT). Slices were subsequently treated with PBS containing 0.2% TX100 (2 h, RT), blocked with PBS containing 0.05% TX100 and 10% normal goat serum (NGS, 2 h, RT), and incubated overnight (4°C) with anti-TAT antibody (1/50 dilution, Cell Signaling Technology, #2547S) in PBS containing 5% NGS and 0.05% TX100. The following day, slices were washed with PBS and incubated for 2 h at room temperature with secondary antibody (1/200 anti-mouse IgG linked to Alexa 488, Invitrogen Life Sciences) in PBS containing 5% NGS and 0.05% TX100. Slides were then rinsed with PBS and nuclei were counterstained with DAPI (1 μg/mL). Finally, slides were mounted in Vectashield mounting medium (Vector Labs). Images were acquired using a BD Pathway BioImaging System (Becton Dickinson) that includes stitching software, obtaining a montage image of the entire brain section. Intensity of Alexa 488 fluorescence in 10 analysis zones in the left and right cortex respectively was analyzed using ImageJ software.

### TAT-Gap19 peptide

TAT-Gap19 (YGRKKRRQRRR-KQIEIKKFK) was synthesized by Pepnome Inc. (Hongkong, China) at a purity of 95%. It was dissolved in PBS and aliquoted/stored as a stock solution of 10 mM.

### Data analysis and statistics

The data are expressed as mean ± s.e.m., with “n” denoting the number of independent experiments. Two groups were compared by student's *t*-test and two-tail *p*-value. In experiments where the effects of different treatments were assessed on normalized data, non-parametric ANOVA Kruskal-Wallis tests were performed. Tests with significance of *p* < 0.05 were followed by a Dunn's multiple comparison *post-hoc* test using the GraphPad Prism version 5.00 (San Diego, CA, USA). Unless stated otherwise, significance as compared to control condition based on the raw data (before normalization) was assessed by two-tailed Wilcoxon signed-ranked tests. The level of significance was set at *p* < 0.05. Graphics were prepared using Microcal Origin 6.0 (Northampton, MA, USA) and Adobe Illustrator 10 (San Jose, CA, USA).

## Results

Recently, it was reported that Gap19 (KQIEIKKFK; see Figure 1) inhibits Cx43 hemichannel activity but not gap junctional communication in the heart (Wang et al., 2013b). To determine whether these findings also apply for brain cells, especially astrocytes which predominantly express Cx43 (Ransom and Giaume, 2013), we tested the effect of this peptide on hemichannel activity (Etd^+^ uptake and ATP release assays) and gap junctional communication (scrape loading and dye transfer) in two *in vitro* preparations: primary cultures of astrocytes and acute hippocampal slices.

**Figure 2:**
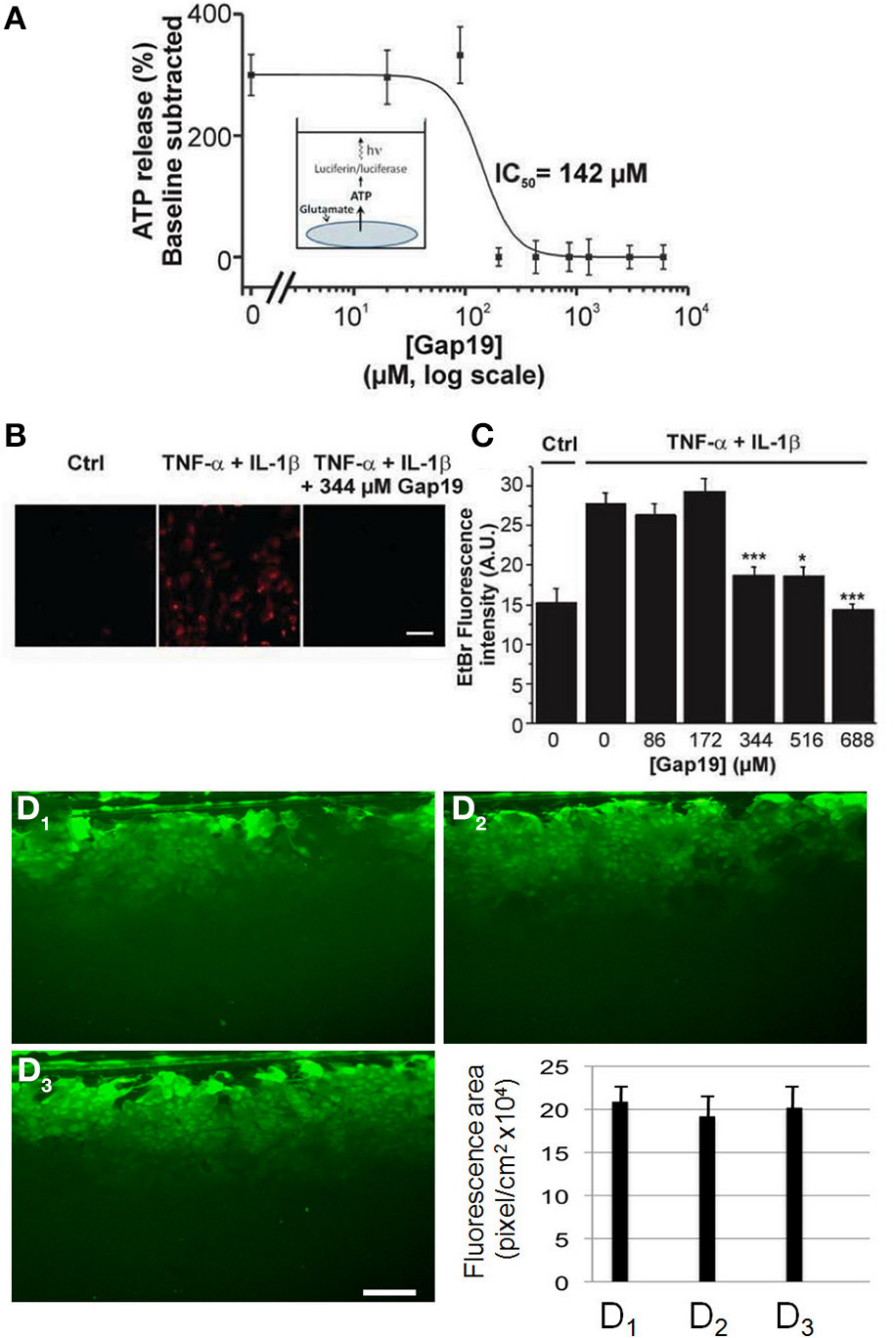
**Dose-dependent inhibition of ATP release and Etd^+^ uptake through Cx43 hemichannels by Gap19 with lack of effect on gap junctional communication in cultured astrocytes**. **(A)** Concentration-dependent inhibition by Gap19 (30 min pre-incubation) of ATP release in cultured cortical astrocytes triggered by glutamate (100 μM, 15 min application) (*n* = 6 independent experiments). **(B)** Representative images showing Etd^+^ uptake (red) in cultured astrocytes under control conditions (Ctrl) and after TNF-α/IL-1β or TNF-α/IL-1β + Gap19 treatment. Scale bar: 20 μm. (^*^p < 0.05; ^***^p < 0.001). **(C)** Summary data of Etd^+^ uptake studies in astrocytes, demonstrating inhibition by Gap19 (*n* = 5–8 independent experiments). Statistical comparisons refer to the stimulus condition without Gap19 (zero Gap19 concentration). (**D_1_–D_3_**) Representative images of scrape-loading dye transfer experiment in confluent cultures of astrocytes. Compared to control condition **(D_1_**), with Gap19 344 μM (**D_2_**, 30 min pre-incubation) or 688 μM (**D_3_**, 30 min pre-incubation). Lower graph: Quantification of scrape-loading data indicating that Gap19 did not influence gap junctional coupling as measured in confluent cultures of astrocytes (from left to right, bars are from control and the two tested concentrations of Gap19, respectively). (*n* = 3–5 independent experiments).

While *in vivo* astrocytes express two major Cxs, Cx43 and Cx30, astrocytes in primary culture offer the advantage that only one of them is expressed, namely Cx43 (Giaume et al., 1991a). Indeed, Cx30 is detected in astrocytes only either after 10–11 weeks of solo-culture (Kunzelmann et al., 1999) or in 3 week-old astrocytes after 1 week of co-culture with neurons (Koulakoff et al., 2008). Firstly, we tested whether Gap19 influences hemichannel activity in primary cultures of astrocytes stimulated by glutamate (100 μM, 15 min), a treatment that has been reported to trigger hemichannel-mediated ATP release in primary astrocytes (De Vuyst et al., 2009). As illustrated in Figure 2, we found that Gap19 (30 min treatment) inhibited glutamate-triggered ATP release (Figure 2A). Alternatively, Cx43 hemichannel activity was also monitored by an Etd^+^ uptake assay in astrocytes treated with a combination of two pro-inflammatory cytokines, TNF-α and IL-1β (10 ng/ml for each, 3 h), a procedure that activates Cx43 hemichannels in cultured astrocytes (Retamal et al., 2007). Under those conditions, we observed that Etd^+^ uptake was inhibited in the presence of Gap19 in a dose-dependent manner, with the peptide applied prior to (30 min) and during Etd^+^ uptake (Figures 2B,C). Finally, since in confluent cultures of astrocytes intercellular communication through Cx43 gap junction channels is high (Giaume et al., 1991b), we tested whether Gap19 (344 μM and 688 μM 30 min) had any effect on the level of gap junctional coupling. Experiments performed with the scrape loading and dye transfer technique demonstrated that Gap19 was without any effect on gap junctional communication (Figures 1D_1_–D_3_ and summary bar chart) in astrocytes which in culture express only Cx43 (see Koulakoff et al., 2008).

**Figure 3:**
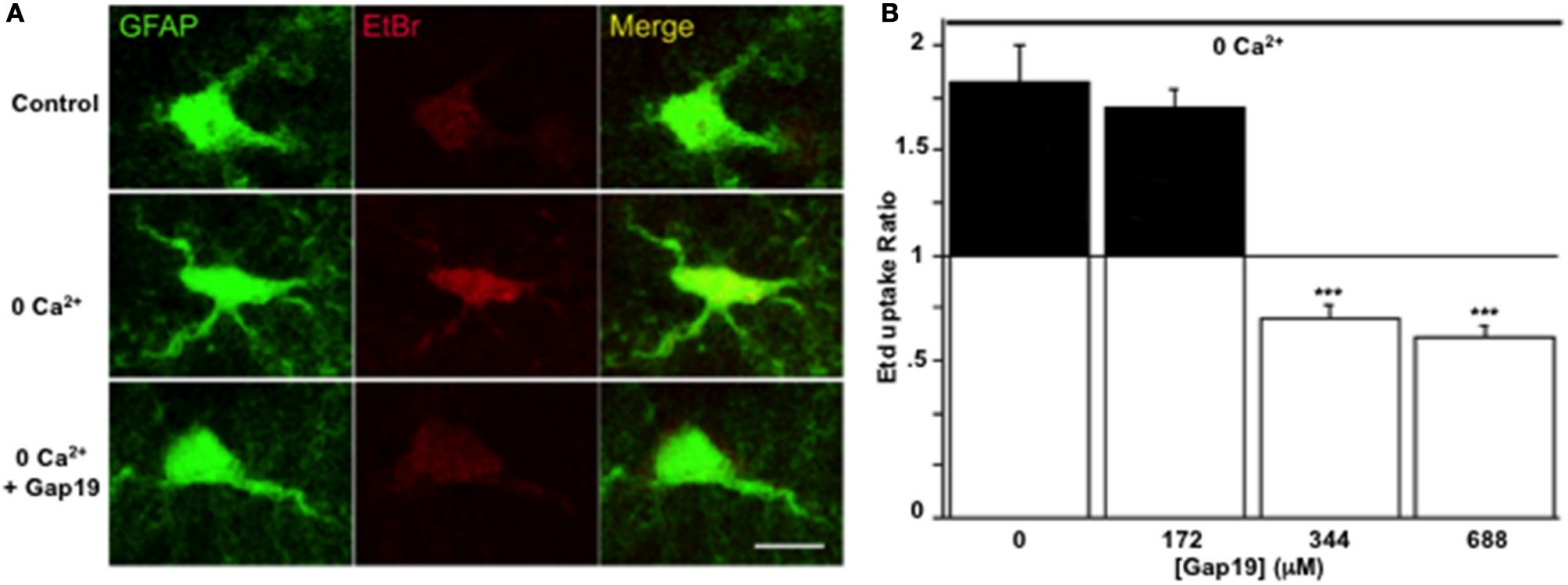
**Gap19 inhibits hemichannel activity in astrocytes studied in acute hippocampal slices**. **(A)** Representative images of Etd^+^ uptake (red) in astrocytes (green) in hippocampal slices from GFAP-eGFP transgenic mice, under control conditions (upper row) and after 10 min exposure to a Ca^2+^-free solution without (middle row) or with (lower row) 344 μM Gap19 treatment. Scale bar: 10 μm. **(B)** Summary graph demonstrating significant Etd^+^ uptake in astrocytes that was inhibited by Gap19 used at concentration of 344 and 688 μM. Statistical comparisons in **(B)** were done with the stimulus condition without Gap19 (*n* = 3 independent experiments; ^***^p < 0.001).

The inhibitory effect of Gap19 *in situ* was then tested by performing the Etd^+^ uptake assay in acute hippocampal brain slices from GFAP-eGFP transgenic mice. While in normal saline solution Etd^+^ uptake was very weak, as reported previously (Orellana et al., 2011), treatment of the slices with a Ca^2+^-free solution (no added Ca^2+^ and 5 mM of the Ca^2+^-buffer EGTA), a condition known to activate Cx43 hemichannels in astrocytes (Ye et al., 2003) and that can be inhibited by carbenoxolone in hippocampal slices (Rouach et al., 2008), induced Etd^+^ uptake in GFP-positive cells. This increase in Etd^+^ uptake was not inhibited by Gap19 (applied 30 min prior to and during Etd^+^ uptake) used at 172 μM while concentrations of 344 and 688 μM inhibited the uptake of Etd^+^ by GFP-positive cells (Figures 3A,B).

**Figure 4:**
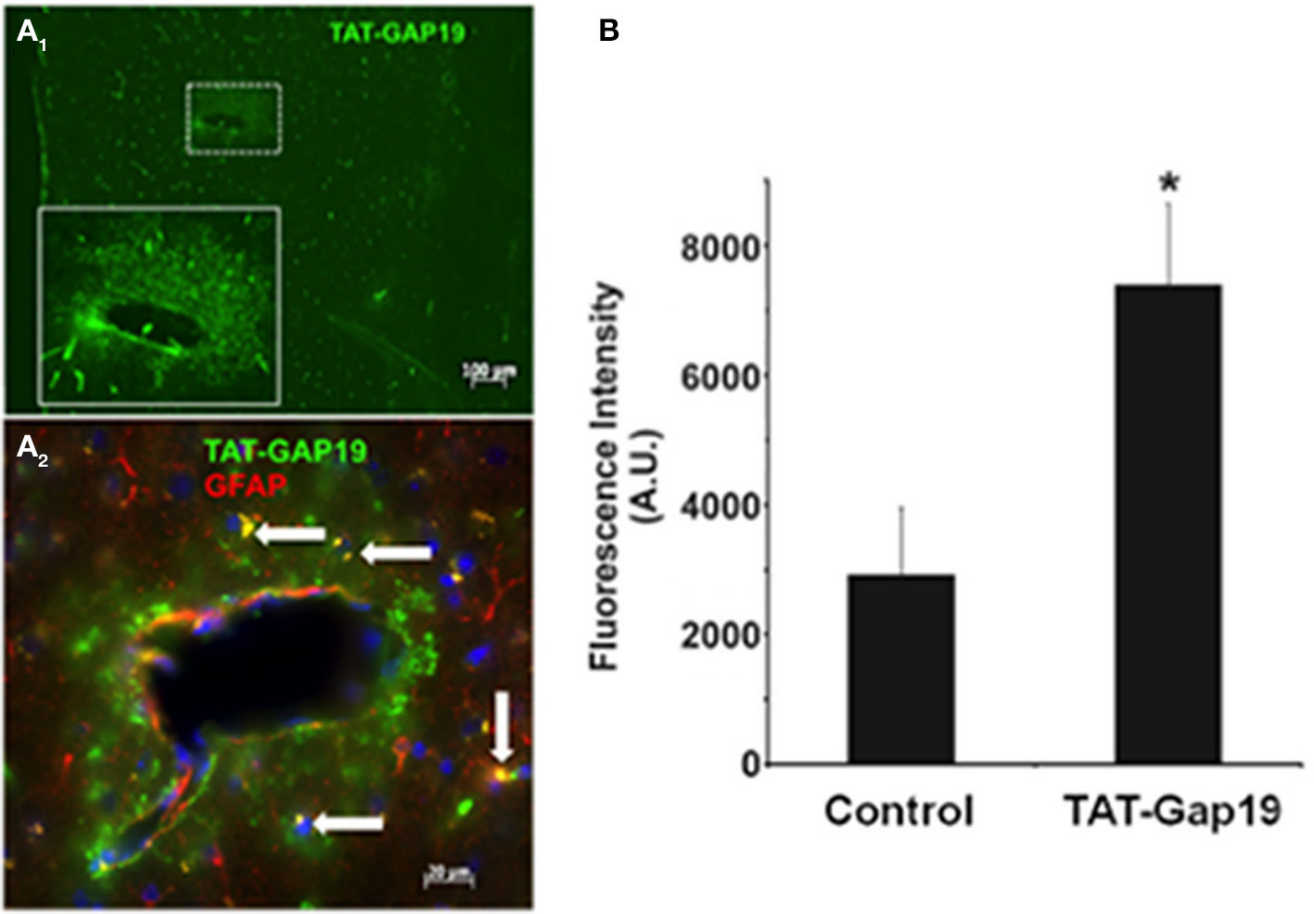
**Detection of TAT-Gap19 in the cortex of the mouse. (A_1_–A_2_)** One hour after carotid injection of TAT-Gap19 the brain displayed clear TAT immunoreactivity compared to mice that received vehicle (PBS) only. (**A_1_**), Area in small box is enlarged in the lower right box of the panel; **(A_2_)**, Double labeling with anti-TAT and anti-GFAP antibodies indicates that some GFAP-positive astrocytes (white arrows) have taken up the TAT peptide. Note that the green fluorescence is concentrated in the white box area probably because this represents a large vessel that would have more passage of TAT-Gap19 into the brain parenchyma surrounding the vessel. **(B)** A single i.v. injection of TAT-Gap19 gave significant immune signal in the brain 24 h later. Taken together, these experiments indicate that TAT-Gap19 traverses the blood-brain barrier and is retained in the cells. Fluorescence intensities of Alexa 488 signal of the secondary antibody determined in slices immunostained with a primary antibody directed against the TAT sequence. Control represents experiments in mice injected with PBS vehicle (*n* = 5 for control and TAT-Gap19; ^*^p < 0.05).

The possibility of *in vivo* application of hemichannel blockers was explored through peripheral administration of Gap19 linked to the TAT membrane translocation motif at its N-terminal end (TAT-Gap19) in adult mice. One hour after carotid injection (45 mg/kg), animals were sacrificed and the brain tissue prepared for immunohistochemistry to localize TAT-Gap19 making use of an antibody directed against the TAT sequence. Figure 4A shows that following this treatment, TAT immunoreactivity was readily detected throughout the brain as compared to the brain tissue of mice that received the vehicle (PBS) only. Gap19 has an intracellular target located on the C-terminal tail of Cx43 to which it binds with a K_d_ of ~2.5 μM (Wang et al., 2013b), making it possible that the peptide can be trapped and retained intracellulary. We therefore tested whether administration of a single dose of TAT-Gap19 i.v. (55 mg/kg, giving an estimated ~250 μM concentration assuming distribution in the blood volume) resulted in detectable peptide signal in the brain 24 h after administration, again based on TAT immunostaining. Figure 4B illustrates the average data of such experiments, demonstrating that the TAT-Gap19 signal was significantly above the background level, indicating that TAT-Gap19 was retained in the brain tissue.

## Discussion

Gap19 is a peptide that corresponds to a sequence on the cytoplasmic loop (CL) of Cx43. Its sequence is part of the L2 domain that is involved in interactions of the CL with the CT-tail of Cx43. Interestingly, CT-CL interaction has a differential effect on hemichannels and gap junction channels: it closes gap junction channels while it is necessary for hemichannel opening (Ponsaerts et al., 2010; D'hondt et al., 2013; Iyyathurai et al., 2013; Wang et al., 2013a,b). This differential effect is most probably the consequence of distinct channel configurations of hemichannels as compared to gap junction channels (Wang et al., 2013b). Gap19 peptide binds to the CT and thereby prevents interaction of the CT with the CL, making hemichannels unavailable for opening (Wang et al., 2013a). Gap19 has most extensively been characterized in C6 cells overexpressing Cx43 and in cardiomyocytes (Wang et al., 2013a). We here demonstrate that Gap19 also inhibits hemichannel activity in astrocytes while not affecting gap junctional coupling. The concentrations necessary to achieve hemichannel block in astrocytes appeared to be somewhat higher than in Cx43-expressing C6 cells: in the latter, half-maximal inhibition of hemichannel opening triggered by exposure to the Ca^2+^-ionophore A23187 occurred at ~47 μM (Wang et al., 2013b) while the half-maximal effect concentration was ~142 μM for glutamate-triggered ATP release in astrocyte cultures (Figure 2A) and in the 250 μM range when hemichannels were opened with TNF-α/IL-1β in astrocyte cultures or with zero extracellular Ca^2+^ in brain slices (Figure 3B). These differences may relate to differences in the triggers used to induce hemichannel opening (increase of intracellular Ca^2+^ vs. decrease of extracellular Ca^2+^ or cytokine exposure) or differences in the hemichannel assays used (patch clamp, ATP release, dye uptake). Gap19 needs to enter the cell in order to reach its target located on the CT-tail of Cx43; it is endowed with some intrinsic plasma membrane-permeability that is related to the fact that 4 AAs of the nonapeptide are positively charged Lys residues. However, linking Gap19 to the HIV-derived TAT internalization sequence further promotes its membrane permeability and reduces the concentration for half-maximal inhibition of Cx43 hemichannels in C6 cells from ~47 μM to ~7 μM (Wang et al., 2013b), Supplemental Data. This 7 μM concentration is in good agreement with the ~6.5 μM half-maximal inhibition of unitary Cx43 hemichannel currents when Gap19 is dialyzed in the cell via the patch pipette. Finally, the effects of Gap19 on hemichannel activity reported here are similar to those previously described for Gap26 and Gap27 that also blocks hemichannel activity in astrocytes induced by LPS or pro-inflammatory treatments in culture astrocytes (see Retamal et al., 2007; Froger et al., 2010) and in acute brain slices treated with the amyloid peptide (Orellana et al., 2011). We have also published that carbenoxolone blocks hemichannel activity induced by a calcium free solution (Rouach et al., 2008, Supplementary Data Figure S5).

The *in vivo* injection experiments demonstrate that TAT-Gap19 can be detected in the brain tissue, based on immunostaining for the TAT moiety of the peptide (Figure 4). This observation indicates that TAT-Gap19 can successfully cross the blood-brain barrier, which is expected as a result of the presence of the TAT membrane translocation motif. We did not quantify the immune signal in terms of estimates of the concentration attained. However, the fact that there is still significant TAT-Gap19 immune signal 24 h after a single i.v. injection indicates that the peptide is retained for several hours in the cells, which is plausible given the fact that the K_d_ for Gap19 interaction with its target on the CT-tail is in the micromolar range (~2.5 μM). Finally, double labeling with anti-TAT and anti-GFAP antibodies indicates that the peptide reaches GFAP-positive astrocytes, however, several TAT-positive cells are apparently not GFAP-positive. This is not surprising for experiments performed in the cortex of adult mice where it is well known that only a few astrocytes are GFAP-positive at this age (see for instance Nolte et al., 2001; Houades et al., 2006). Consequently, we cannot exclude that the TAT-peptide staining could also come from endothelial cells or microglia that have both been reported to express Cx43 (Orellana et al., 2011).

The L2 peptide, from which Gap19 is derived, is also a specific hemichannel blocker (Ponsaerts et al., 2010) but Gap19 has the advantage that it is smaller and contains the most crucial motif to engage in interactions with the target(s) on the CT-tail. Stehberg and collaborators have used TAT-L2 for local injection in the amygdala in *in vivo* animal studies on fear memory (Stehberg et al., 2012). Their work demonstrated that fear memory was suppressed by TAT-L2 and was rescued upon addition of a cocktail of several proposed gliotransmitter substances, suggesting a role of gliotransmitter release via astrocytic hemichannels. Of note, both L2 and Gap19 (or their TAT-linked versions) are specific not only for hemichannels but also for the Cx43 protein. The CL and CT domains are very different between different connexin species and in line with this, we found that Gap19 does not affect Cx40 hemichannels (Wang et al., 2013a). Moreover, Gap19 does not block Panx1 channels (Wang et al., 2013a), making Gap19 a potentially powerful tool to decipher the role of astroglial Cx43 hemichannels in brain functions and pathologies (see Giaume et al., 2013).

### Conflict of interest statement

The reviewer Dr. Retamal declares that, despite having collaborated with the author Dr. Giaume, the review process was handled objectively. The authors declare that the research was conducted in the absence of any commercial or financial relationships that could be construed as a potential conflict of interest.
